# Aortic arch calcification and risk of cardiovascular or all-cause and mortality in dialysis patients: A meta-analysis

**DOI:** 10.1038/srep35375

**Published:** 2016-10-17

**Authors:** Ao Zhang, Shiji Wang, Hongxiang Li, Juan Yang, Hui Wu

**Affiliations:** 1 Department of Intensive Care Unit, First Hospital of Jilin University, Changchun 130021, China; 2 Department of Ophthalmology, First Hospital of Jilin University, Changchun 130021, China

## Abstract

Studies on aortic arch calcification (AAC) and mortality risk in maintenance dialysis patients have yielded conflicting findings. We conducted this meta-analysis to investigate the association between the presence of AAC and cardiovascular or all-cause and mortality risk in maintenance dialysis patients. Observational studies evaluating baseline AAC and cardiovascular or all-cause mortality risk in maintenance dialysis patients were searched through the PubMed and Embase, CNKI, VIP and Wanfang databases until January 2016. A total of 8 studies with 3,256 dialysis patients were identified. Compared with patients without AAC, the presence of AAC was associated with greater risk of cardiovascular mortality (hazard risk [HR] 2.30; 95% confidence intervals [CI] 1.78–2.97) and all-cause mortality (HR 1.44; 95% CI 1.19–1.75). Subgroup analyses indicated that the pooled HR for cardiovascular and all-cause mortality was 2.31 (95% CI 1.57–3.40) and 1.45 (95% CI 1.08–1.96) for the grade 2/3 AAC. Peritoneal dialysis patients with AAC had greater cardiovascular (HR 3.93 *vs.* HR 2.10) and all-cause mortality (HR 2.36 vs. HR 1.33) than hemodialysis patients. The AAC appears to be independently associated with excessive cardiovascular and all-cause mortality in maintenance dialysis patients. Regular follow-up AAC might be helpful to stratify mortality risk in dialysis patients.

Cardiovascular disease is the most common causes of death in patients with end-stage kidney disease who are undergoing maintenance dialysis. Vascular calcification is very frequent finding in the dialysis patients^1^,^2^. Vascular calcification in the aorta and coronary arteries have been recognized as an important risk factor for adverse outcomes in dialysis patients^1^.

Chest radiography is a routine screening test performing on dialysis patients. Aortic arch calcification (AAC) measurement by the chest radiography was strongly associated with cardiovascular events in the general population^2^,^3^. AAC predicted the renal function decline in patients with stage 3 to 5 chronic kidney diseases^4^. In dialysis patients, the presence of AAC was significantly associated with increased risk of mortality^5^,^6^,^7^,^8^. However, data derived from chest radiography analysis on the presence or grade of AAC and cardiovascular or all-cause mortality in dialysis patients remain controversial^5^,^6^,^7^,^9^,^10^. In addition, the magnitude of risk estimates varied obviously across studies. The discrepancy may be correlated with differences in patient characteristics and calcification.

We therefore conducted a meta-analysis of the available observational studies to determine the presence and severity of AAC and cardiovascular or all-cause mortality in dialysis patients.

## Results

### Selected studies and characteristics

**Figure 1:**
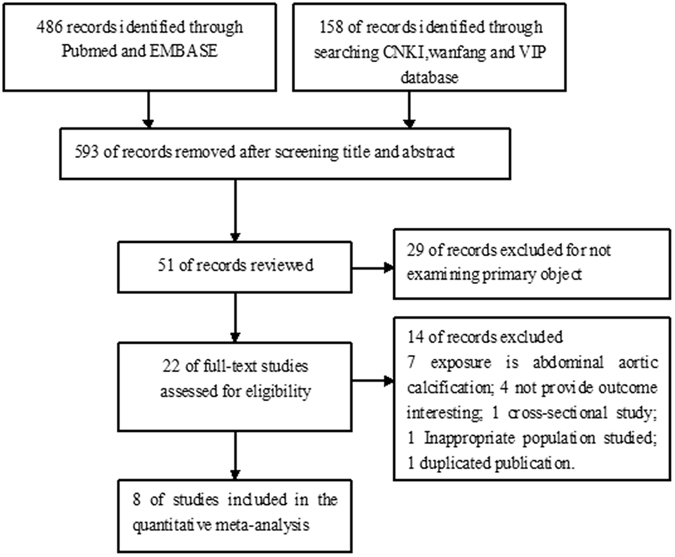
Flow chart of the study selection process.

We initially retrieved 644 studies through electronic searches. After scanning the title and abstract, 593 articles were removed mainly because they were reviews, meeting abstract or not relevant outcomes reported. After applying our predefined inclusion criteria, 43 studies were removed mainly due to they did not provide outcome interesting or exposures were abdominal aortic calcification. Finally, eight studies^5^,^6^,^7^,^8^,^9^,^10^,^11^,^12^ satisfied the inclusion criteria, providing data on 3,256 dialysis patients. Flow chart of study selection process is detailed in Fig. 1.

Table 1 summarizes the baseline characteristics of the included studies. The included studies were published from 2010 to 2015. The follow-up duration ranged from 1.8 to 10 years. Five studies^5^,^6^,^8^,^9^,^11^ were prospective design and three studies^7^,^10^,^12^ were retrospective design. Among the 8 studies, 2 articles^10^,^12^ were conducted in America and 6 articles^5^,^6^,^7^,^8^,^9^,^11^ in Asia. All the studies determined the AAC using plain chest X-rays image. The prevalence of AAC in dialysis patients varied from 40.7% to 58%. All the included studies reported all-cause mortality as the outcome and six studies reported cardiovascular mortality. On the basis of NOS for cohort studies, the NOS scores of the included studies ranged from 5 to 8 stars.

### All-cause and cardiovascular mortality

**Figure 2:**
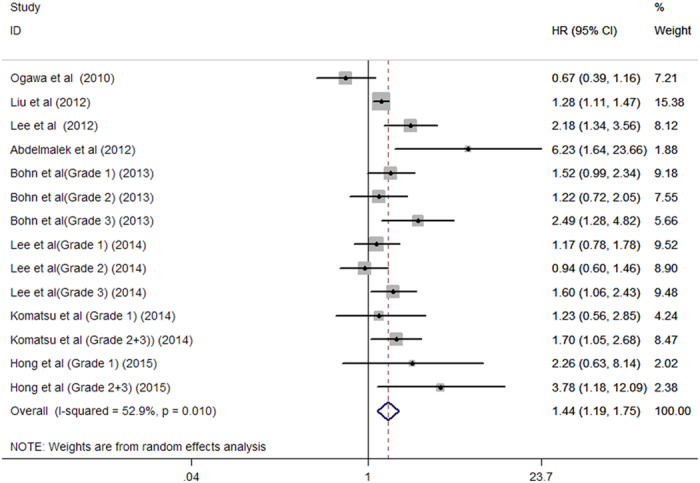
Forest plots showing HR and 95% CI of all-cause mortality compared with and without aortic arch calcification in a random effect model.

**Figure 3:**
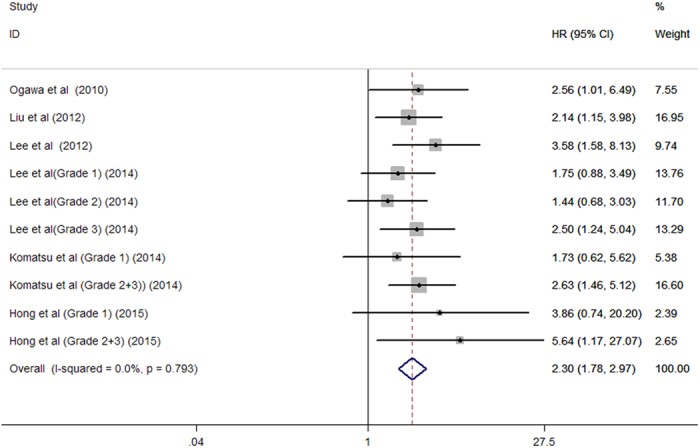
Forest plots showing HR and 95% CI of cardiovascular mortality compared with and without aortic arch calcification in a fixed-effect model.

A total of 766 all-cause mortality cases were reported in eight studies^5^,^6^,^7^,^8^,^9^,^10^,^11^,^12^ among 3,256 dialysis patients. As shown in Fig. 2, the presence of AAC was associated with 44% greater risk of all-cause mortality (HR 1.44; 95% CI 1.19–1.75; I^2^ = 52.9%; *P* = 0.010) in a random effect model. A total of 287 cardiovascular mortality cases were reported in six studies^5^,^6^,^7^,^8^,^9^,^11^ among 2,339 dialysis patients. As shown in Fig. 3, the presence of AAC was associated with 1.30 folds greater risk of cardiovascular mortality (HR 2.30; 95% CI 1.78–2.97; I^2^ = 0.0%; *P* = 0.793) in a fixed-effect model.

### Subgroup analyses and sensitivity analyses

Subgroup analyses based on study design, region, patient population sample sizes, grade of AAC, and follow-up duration showed similar results across all the analyses (Table 2). Sensitivity analyses by excluding any single study at each turn indicated that there were no changes in the direction of pooling risk estimate of all-cause mortality (pooled HR ranges from 1.39 to 1.57) and cardiovascular mortality (pooled HR ranges from 2.19 to 2.63).

### Publication bias

**Figure 4:**
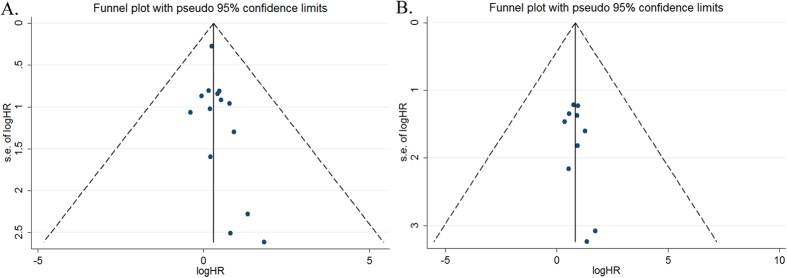
Funnel plot showing publication bias based on the all-cause mortality (**A**) and cardiovascular mortality (**B**).

Evidences of publication bias for all-cause mortality were not observed based on the funnel plot (Fig. 4A), Begg’s rank correlation test (*P* = 0.101), and Egger’s linear regression test (*P* = 0.134). There were also no evidences of publication bias for cardiovascular mortality according to the funnel plot (Fig. 4B), Begg’s rank correlation test (*P* = 0.371) and Egger’s linear regression test (*P* = 0.207).

## Discussion

The present meta-analysis provided evidences that the presence of AAC significantly increased the risk of all-cause mortality by 44% and cardiovascular mortality by 130% in dialysis patients. To the best of our knowledge, this is the first meta-analysis to investigate the relationship between the presence and severity of AAC and risk of cardiovascular and all-cause mortality in dialysis patients. Given AAC is easily determined by chest X-ray in clinical practice, regular follow-up AAC might be a simple and helpful method to stratify the mortality risk in dialysis patients.

Subgroup analysis revealed that the statistical significance of an association with mortality was more obvious in patients with grade 2 and 3 AAC. This finding supports a higher degree of AAC corresponds to a greater mortality risk. In addition, the presence of AAC in peritoneal dialysis patients appeared to have a greater mortality risk than those undergoing hemodialysis patients. Moreover, progression of AAC over one year was also an independent predictor of cardiovascular and all-cause mortality in incident peritoneal dialysis patients^8^.

Approximately 20–30% of people older than 65 years had calcification in the aorta^2^. In dialysis patients, the prevalence of AAC ranged from 37.29% to 58% based on the chest X-ray findings^7^,^12^. The high prevalence of AAC in dialysis patients indicates the importance to early detect the presence and progression of AAC. Several potential explanations may explain the presence and progression of AAC and mortality risk. AAC represented the magnitude of whole aortic calcification in the general population and dialysis patients^13^,^14^. The extent of AAC may be correlated to the degree of atherosclerosis. Calcification can increase stiffness and reduce elasticity of large arteries, resulting in substantial mortality^15^.

Our meta-analysis had several limitations. First, the most important concern is the sensitivity for detecting AAC in chest X-ray. Compared with plain X-ray, multi-detector computed tomography or electron beam-computed tomography are the gold standard for evaluating AAC, with the power of detecting small amounts of calcification. However, these examinations are too expensive to perform in all the dialysis patients. AAC assessed by a chest X-ray may underestimate the true calcium deposition in the aortic wall. Second, most dialysis patients in our analysis were adult and elder with a trend of acceleration of vascular calcification. Thus, predictive values of AAC on mortality risk cannot be extrapolated to relatively younger dialysis patients. Third, the included studies did not adjust covariates in a consistent way, lacking adjustment for these covariates may have led to a slight overestimation of the risk estimate. Finally, Despite we made a comprehensive literature search, there were very few studies included in this meta-analysis.The conclusion based on the limited number of study may be not robust, particularly in the subgroup analyses.

In conclusion, this meta-analysis indicates that AAC appears to be independently associated with greater risk of cardiovascular and all-cause mortality, and higher grade of AAC corresponds to a greater risk in dialysis patients. Our finding support incorporation of AAC into the existing risk factors for dialysis patients may improve the prognostic stratification. However, more well-designed prospective studies are need to confirm our findings because there were very few studies being included in the meta-analysis.

## Methods

### Search strategy

This meta-analysis was performed in accordance with the recommendations of the Preferred Reporting Items for Systematic Reviews and Meta-analyses Statement^16^ An extensive electronic database search was conducted in PubMed, Embase, China National Knowledge Infrastructure, VIP and Wanfang databases up to January 2016. The following search terms were used: ‘hemodialysis’ OR ‘haemodialysis’ OR ‘peritoneal dialysis’ OR ‘end stage renal disease’ AND ‘aortic calcification’ OR ‘aortic arch’ AND ‘calcification’ OR ‘calcium’ AND ‘mortality’ OR ‘death’ AND ‘follow-up’ OR ‘longitudinal’. Additionally, the reference lists of the selected papers were manually searched for additional possible studies.

### Selection criteria

Studies were considered eligible for the present meta-analysis if: 1) original observational studies; 2) participants in the end-stage kidney disease who are undergoing maintenance dialysis; 3) investigating the relationship between the presence and extent of AAC at baseline and subsequent cardiovascular or all-cause mortality risk; and 4) reporting risk estimate of cardiovascular or all-cause mortality events. The severity of calcification was classified as grade 0 to 3 in accordance with previous studies^6^,^13^,^14^. For the multiple articles from the same research group, we only selected the most recent comprehensive publication. Studies were excluded if they were cross-sectional design, reviews or duplicated publication.

### Data extraction and quality assessment

Two authors (A Zhang and SJ Wang) independently collected data from included studies using a structured form. Extracted information included first author’s name, publication year, study design, geographical region of study, baseline characteristics of patients, detection methods, prevalence of AAC, event numbers, fully adjusted risk ratio (RR) or hazard ratio (HR) and 95% confidence intervals (CI), duration of follow-up, and adjustment for covariates. Any discrepancies during the data extraction were resolved by discussion. We applied the Newcastle–Ottawa Scale (NOS) for cohort studies to evaluate the methodological quality of each study^10^. The NOS ranges from zero to nine stars. Studies achieving a rating of more than 6 stars were considered to be of higher quality.

### Statistical analysis

The overall risk estimates were pooled using the most fully adjusted RR or HR with their 95% CI comparing with and without AAC. Heterogeneity between studies evaluated by the Cochran’s Q (heterogeneity was set at a value of p < 0.10) and I^2^ tests (I^2^ > 50%). Random effect model was used for meta-analysis when there was significant heterogeneity; otherwise, a fixed-effect model was applied^17^. Subgroup analyses were performed by study design (prospective *vs*. retrospective), region (America *vs*. Asia), population (hemodialysis *vs*. peritoneal dialysis), sample sizes (>500 *vs*. <500), grade of AAC, follow-up duration (≥4 years *vs*. <4 years), and NOS scores (≥6 stars *vs*. <6 stars). The possibility of publication bias was tested by the Begg’s^18^ and Egger’s^19^ tests with significant publication bias considered as a p-value < 0.1.We performed a sensitivity analysis by excluding any single study at each turn to test the robustness of the pooled results. All statistical analyses were conducted using Stata 12.0 software.

## Additional Information

**How to cite this article**: Zhang, A. *et al*. Aortic arch calcification and risk of cardiovascular or all-cause and mortality in dialysis patients: A meta-analysis. *Sci. Rep.* **6**, 35375; doi: 10.1038/srep35375 (2016).

## Supplementary Material

Supplementary Information

## Figures and Tables

**Table 1:** Baseline characteristics of the included studies.

Study/year	Region	Design	Patients (%women)	Age (years)	Detection Methods	Prevalence of AAC	Comparison of AAC	Events NumberRR or HR (95% CI)	Follow-up (years)	Adjustment for Covariates	NOS
Ogawa *et al*.^9^	Japan	Prospective study	HD 401 (32.7)	58 ± 13 (CAC); 65 ± 11 (no CAC)	Plain chest radiography	50.6%	Presence vs. absence	Cardiovascular death (41) 2.56 (1.01–6.49) Total death (72) 0.67(0.39–1.16)	4	Age, DB, BMI, DBP, hemoglobin, serum albumin, Kt/V level, and creatinine	5
Lee *et al*.^8^	Korea	Prospective study	PD 415 (43.7)	55.8 ± 13.8	Posterior- anterior plain chest X-rays	40.7%	Presence vs. absence	Cardiovascular death (39) 3.58 (1.58–8.13) Total death (90) 2.18(1.34–3.56)	2.85	Age, DB, previous CVD, lipid-lowering medication, calcium phosphorus products, Hs-CRP and albumin	7
Liu *et al*.^11^	China	Prospective study	HD 333 (46.5)	52 ± 14	Plain chest radiography	Not provided	Presence vs. absence	Cardiovascular death (59) 2.14(1.15–3.98) Total death (105) 1.28(1.11–1.47)	4.2	Age, gender, dialytic vintage, dialysis modality, DB, blood pressure, hemoglobin, ferritin, CRP and LVMI.	6
Abdelmalek *et al*.^12^	USA	Retrospective study	HD 93 (3)	66 ± 11(CAC); 63 ± 10 (no CAC)	Frontal and lateral chest radiograph	58%,	Presence vs. absence	Total death (26) 6.23(1.64–23.66)	1.8	Age, CAD, pre-dialysis creatinine, phosphorus, DB, hyperlipidemia and CAC.	6
Bohn *et al*.^10^	Canada	Retrospective cohort study	HD 824(46)	59.7	Postero-anterior X-ray	46%	Gr. vs. absence	Total death (152) 1.52(0.99–2.34) Gr. 1 1.22(0.72–2.05) Gr. 2 2.49(1.28–4.82) Gr. 3	3	Age at x-ray, race, sex, duration of dialysis, DB, history of heart failure, IHD, serum phosphate and creatinine at initiation of dialysis.	6
Komatsu *et al*.^6^	Japan	Prospective study	HD 301 (34)	63.8 ± 12.2	Chest X-rays	41.9%	Gr. vs. absence	Cardiovascular death (43) 1.73 (0.62–5.62) Gr. 1 2.63 (1.46–5.12) Gr. 2 + 3 Total death (65) 1.23(0.56–2.85) Gr. 1 1.70(1.05–2.68) Gr. 2 + 3	3	Age, DB, serum albumin, non-HDL TC, hypertension, prescription of active vitamin D3	7
Lee *et al*.^5^	Taiwan	Prospective study	HD 712 (57.0)	55.6 ± 14.3	X-ray films	57%	Gr. vs. absence	Cardiovascular death (87) 1.75(0.88–3.49) Gr. 1 1.44 (0.68–3.03) Gr. 2 2.50(1.24–5.04) Gr. 3 Total death (231) 1.17(0.78–1.78) Gr. 1 0.94(0.60–1.46) Gr. 2 1.60(1.06–2.43) Gr. 3	10	Age, DB, cardiothoracic ratio, albumin, creatinine, non-fasting glucose, phosphorus, calcium phosphorus product, TC, intact parathyroid hormone, alkaline phosphatase	8
Hong *et al*.^7^	China	Retrospective cohort study	HD 177 (41.8)	62.86 ± 14.33	Chest X-rays	37.29%	Gr. vs. absence	Cardiovascular death (18) 3.86 (0.74–20.2) Gr. 1 5.64 (1.17–27.07) Gr. 2 + 3 Total death (25) 2.26(0.63–8.14) Gr. 1 3.78(1.18–12.09) Gr. 2 + 3	2	Age, BMI, albumin, hemoglobin, HDL,LDL, serum phosphate, serum calcium, calcium phosphorus products, and residual renal function	5

Abbreviations: AAC, aortic arch calcification; DB, diabetes; RR, risk ratio; HR, hazard ratio; Gr, grade; NOS, Newcastle–Ottawa Scale; PD, peritoneal dialysis; BMI, body mass index; CAC, coronary artery calcification; CVD, cardiovascular disease; CAD, coronary artery disease; TC,total cholesterol; CRP,C-reactive protein; LVMI, left ventricular mass index; HDL, high-density lipoprotein; DBP, diastolic blood pressure; Hs-CRP, high sensitivity C-reactive protein.

**Table 2:** Subgroup analyses of all-cause and cardiovascular mortality.

Subgroups	Number of studies	Pooled hazard risk	95% confidence interval	Heterogeneity between studies
**1. All-cause** **mortality**				
Study design				
Prospective study	5	1.29	1.05 to 1.59	P = 0.042; I^2^ = 51.8%
Retrospective study	3	1.99	1.33 to 2.99	P = 0.125; I^2^ = 42.1%
Region				
Asia	6	1.35	1.09 to 1.67	P = 0.030; I^2^ = 51.4%
America	2	1.85	1.15 to 2.98	P = 0.082; I^2^ = 55.2%
Patient population				
Hemodialysis	6	1.33	1.10 to 1.62	P = 0.031; I^2^ = 49.5%
Peritoneal dialysis	2	2.36	1.54 to 3.60	P = 0.692; I^2^ = 0.0%
Sample sizes				
>500	2	1.36	1.08 to 1.72	P = 0.195; I^2^ = 32.1%
<500	6	1.59	1.13 to 2.26	P = 0.005; I^2^ = 65.4%
Follow-up duration				
≥4 years	3	1.23	1.09 to 1.38	P = 0.088; I^2^ = 50.5%
<4 years	5	1.78	1.45 to 2.19	P = 0.256; I^2^ = 21.0%
Grade of AAC				
Grade 1	4	1.35	1.03 to 1.77	P = 0.669; I^2^ = 0.0%
Grade 2 + 3	4	1.55	1.13 to 2.12	P = 0.079; I^2^ = 49.3%
**2. Cardiovascular mortality**				
Study design				
Prospective study	5	2.22	1.70 to 2.88	P = 0.809; I^2^ = 0.0%
Retrospective study	1	4.71	1.51 to 14.71	P = 0.744; I^2^ = 0.0%
Region				
Asia	4	2.25	1.66 to 3.06	P = 0.550; I^2^ = 0.0%
America	2	2.42	1.51 to 3.87	P = 0.803; I^2^ = 0.0%
Patient population				
Hemodialysis	4	2.10	1.59 to 2.77	P = 0.893; I^2^ = 0.0%
Peritoneal dialysis	2	3.93	2.02 to 7.64	P = 0.881; I^2^ = 0.0%
Sample sizes				
>500	1	1.86	1.24 to 2.81	P = 0.559; I^2^ = 0.0%
<500	5	2.63	1.90 to 3.65	P = 0.853; I^2^ = 0.0%
Follow-up duration				
≥4 years	3	2.01	1.46 to 2.77	P = 0.8105; I^2^ = 0.0%
<4 years	3	2.91	1.91 to 4.43	P = 0.814; I^2^ = 0.0%
Grade of AAC				
Grade 1	3	1.91	1.10 to 3.30	P = 0.674; I^2^ = 0.0%
Grade 2 + 3	3	2.31	1.57 to 3.40	P = 0.393; I^2^ = 0.0%

AAC, aortic arch calcification.
